# Supplementary material for: Self-Powered Dual-Band Electrochromic Supercapacitor Devices for Smart Window Based on Ternary Dielectric Triboelectric Nanogenerator
Source: Nanomaterials (Basel). 2024 Jan 20;14(2):229. doi: 10.3390/nano14020229 (PMC10820962; doi:10.3390/nano14020229)
Supplement: Supplementary file 1 [file nanomaterials-14-00229-s001.zip › Supporting Information.pdf]

Self-powered Dual-band Electrochromic Supercapacitor Devices for Smart Window Based on Ternary Dielectric Triboelectric Nanogenerator

Tianxiang Zheng [1], Haonan Zhang [1], Chen Chen [1], Xinbo Tu [1], Lin Fang [1], Mingjie Zhang [1], Wen He [1,*] and Peihong Wang [1,2*]

[1] Energy Materials and Devices Key Lab of Anhui Province for Photoelectric Conversion, School of Materials Science and Engineering, Anhui University, Hefei 230601, China; b21301117@stu.ahu.edu.cn (T.Z.); b21101007@stu.ahu.edu.cn (H.Z.); b21301128@stu.ahu.edu.cn (C.C.); b21201056@stu.ahu.edu.cn (X.T.); ms22101018@stu.ahu.edu.cn (L.F.); ms2304163@stu.ahu.edu.cn (M.Z.)

[2] Key Laboratory of Structure and Functional Regulation of Hybrid Materials, Anhui University, Ministry of Education, Hefei 230601, China

* Correspondence: 22169@ahu.edu.cn (W.H.); wangpeihong2002@ahu.edu.cn (P.W.)

Supporting Information

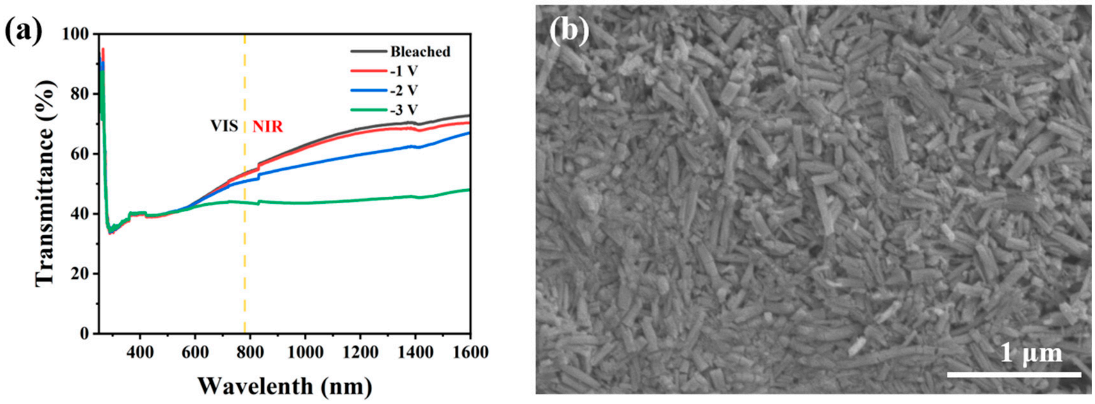

Figure S1. (a) The transmittance spectra of WTO-5 thin film at different voltages in the wavelength range of 350 nm-1600 nm. (b) scanning electron microscopy images of 0.125 M TiO_2/ WO_3 thin film.

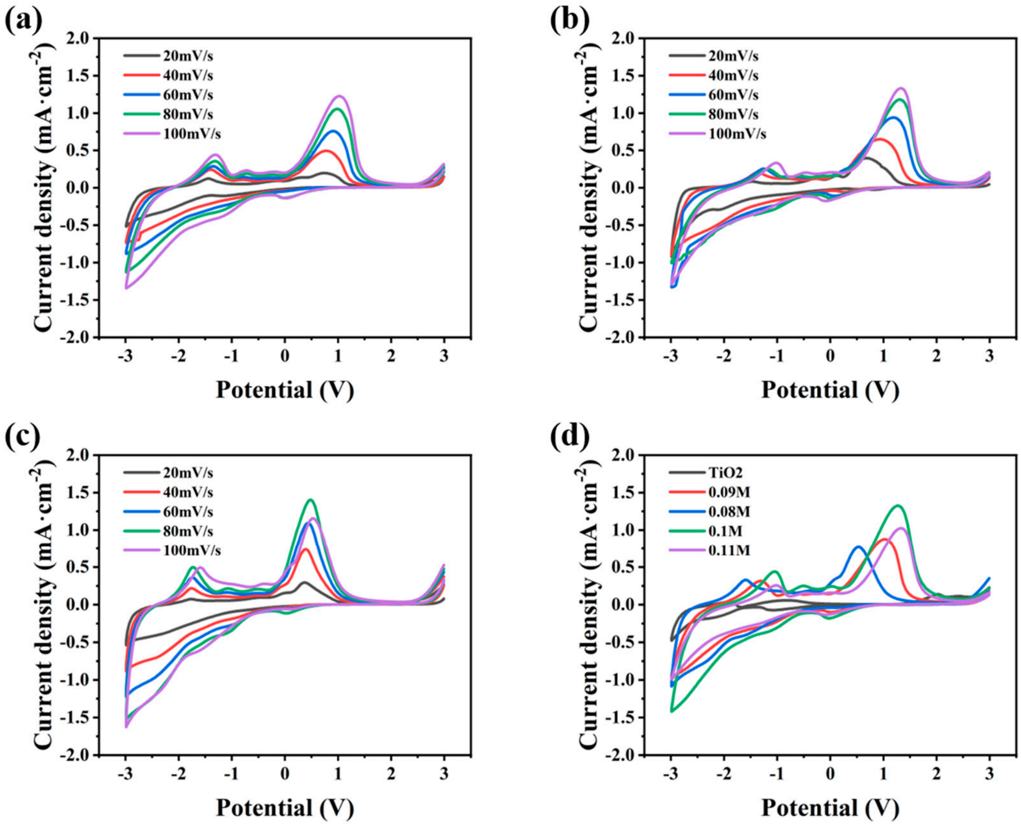

Figure S2. CV curves of the different WTO films at different scan rates (20 to 100 mV • s^{-1}): (a) WTO-1, (b) WTO-2, (c) WTO-4. (d) CV curves of TiO_2 and different WTO films at 100 mV • s^{-1} scan rate.

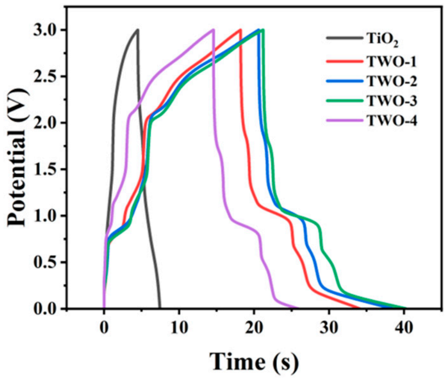

Figure S3. (a) GCD curves of TiO_2 and different WTO films at 0.6 mA • cm^{-1} current density.

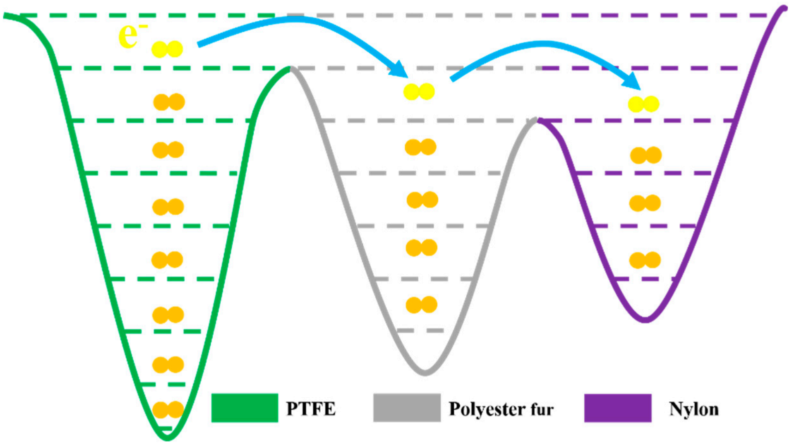

Figure S4. The electron cloud potential well model for surface charge transfer at the surface state among three dielectric materials in contact.

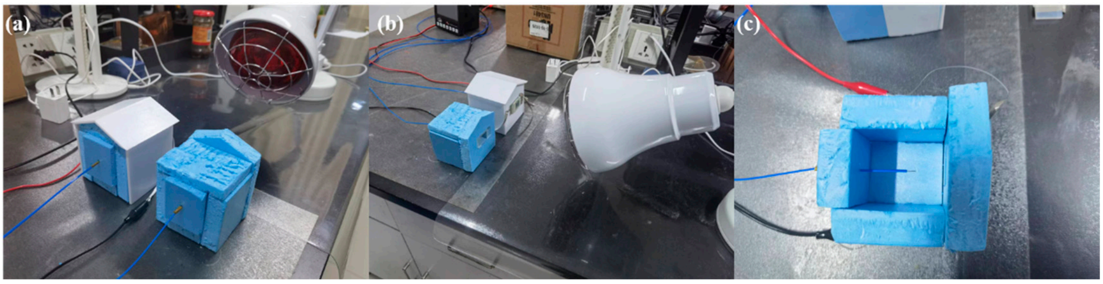

Figure S5. Infrared lamp irradiation experiments in the NIR band. Physical drawing of the test platform: (a) , (b) and (c).
